# THE APOE-ε4 ALLELE AND AGE SYNERGISTICALLY DRIVE DISEASE PROGRESSION IN ALZHEIMER’S DISEASE

**DOI:** 10.1093/geroni/igz038.3427

**Published:** 2019-11-08

**Authors:** Luca Kleineidam, Andrea R Zammit, Alyssa DeVito, Richard B Lipton, Oliver Peters, Alfredo Ramirez, Michael Wagner, Graciela Muniz Terrera

**Affiliations:** 1 Department of Neurodegenererative Diseases and Geriatric Psychiatry, University Hospital Bonn, Bonn, Germany; 2 Department of Neurology, Albert Einstein College of Medicine, Bronx, New York, United States; 3 Department of Psychology, Louisiana State University, Baton Rouge, Louisiana, United States; 5 Department of Psychiatry, Charité Berlin, Berlin, Germany; 6 Department of Psychiatry, University of Cologne, Medical Faculty, Cologne, Germany; 7 Department of Neurodegeneration and Geriatric Psychiatry, University Hospital Bonn, Bonn, Germany; 8 University of Edinburgh, Edinburgh, United Kingdom

## Abstract

The Apolipoprotein E (APOE)-ε4 allele is the strongest genetic risk factor for Alzheimer’s disease (AD) and other neurodegenerative dementias. Cross-sectional case-control studies suggest that the effect of APOE-ε4 decreases in old age. However, since APOE- ε4 is associated with mortality, these studies might be prone to bias due to selective survival. Therefore, we used multi-state-modeling in longitudinal cohort studies to examine the effect of APOE-ε4 on the transition through cognitive states (i.e. cognitively normal, mild cognitive impairment (MCI) and dementia) while taking death as a competing risk into account. Results from the German AgeCoDe study (n=3000, aged 75-101 years) showed that APOE-ε4 increases the risk for cognitive deterioration in all disease stages. Contrary to results from cross-sectional studies, the effect of APOE-ε4 on the transition from MCI to dementia increased with increasing age (HR=1.044, 95%-CI=1.001-1090). The direction of this effect was confirmed in a smaller sample from the Einstein Aging Study (n=744, HR=1.032, 95%-CI=0.949-1.122). To examine the pathophysiological basis of these results, generalized additive models were used to study AD biomarkers in the liquor of 1045 patients with MCI or AD-dementia. Here, increased amyloid (Abeta1-42) pathology was associated with increased tau pathology (pTau181), consistent with the amyloid-cascade-hypothesis. Interestingly, higher age and presence of the APOE-ε4 synergistically lowered the amount of amyloid required to exacerbate tau pathology (interaction p=0.012). Taken together, our results suggest that the effect of APOE-ε4 on disease progression increases with advancing age. An altered neuroinflammatory response to neurodegeneration should be further explored as potential underlying mechanism.

